# Comparison of anchorage reinforcement with temporary anchorage devices or a Herbst appliance during lingual orthodontic protraction of mandibular molars without maxillary counterbalance extraction

**DOI:** 10.1186/s13005-015-0079-4

**Published:** 2015-06-20

**Authors:** Rebecca Metzner, Rainer Schwestka-Polly, Hans-Joachim Helms, Dirk Wiechmann

**Affiliations:** Department of Orthodontics, Hannover Medical School, Hannover, Germany; Department of Medical Statistics, University Medical Center Göttingen, Göttingen, Germany; Private Practice, Bad Essen, Germany

**Keywords:** Orthodontic molar protraction, Anchorage reinforcement, TAD, Herbst appliance, Lingual orthodontics

## Abstract

**Background:**

Orthodontic protraction of mandibular molars without maxillary counterbalance extraction in cases of aplasia or extraction requires stable anchorage. Reinforcement may be achieved by using either temporary anchorage devices (TAD) or a fixed, functional appliance. The objective was to compare the clinical effectiveness of both methods by testing the null-hypothesis of no significant difference in velocity of space closure (in mm/month) between them. In addition, we set out to describe the quality of posterior space management and treatment-related factors, such as loss of anchorage (assessed in terms of proportions of gap closure by posterior protraction or anterior retraction), frequencies of incomplete space closure, and potential improvement in the sagittal canine relationship.

**Methods:**

Twenty-seven subjects (15 male/12 female) with a total of 36 sites treated with a lingual multi-bracket appliance were available for retrospective evaluation of the effects of anchorage reinforcement achieved with either a Herbst appliance (n_subjects_ = 15; 7 both-sided/8 single-sided Herbst appliances; n_sites_ = 22) or TADs (n_subjects_ = 12; 2 both-sided; 10 single-sided; n_sites_ = 14). Descriptive analysis was based on measurements using intra-oral photographs which were individually scaled to corresponding plaster casts and taken on insertion of anchorage mechanics (T1), following removal of anchorage mechanics (T2), and at the end of multi-bracket treatment (T3).

**Results:**

The null-hypothesis was rejected: The rate of mean molar protraction was significantly faster in the Herbst-reinforced group (0.51 mm/month) than in the TAD group (0.35). While complete space closure by sheer protraction of posterior teeth was achieved in all Herbst-treated cases, space closure in the TAD group was achieved in 76.9 % of subjects by sheer protraction of molars, and it was incomplete in 50 % of cases (mean gap residues: 1 mm). Whilst there was a deterioration in the canine relationship towards Angle-Class II malocclusion in 57.14 % of space closure sites in TAD-treated subjects (indicating a loss of anchorage), an improvement in canine occlusion was observed in 90.9 % of Herbst-treated cases.

**Conclusion:**

Subjects requiring rapid space closure by molar protraction in combination with a correction of distal occlusion may benefit from using Herbst appliances for anterior segment anchorage reinforcement rather than TAD anchorage.

## Introduction

The need for rational space management in subjects with missing posterior teeth is a clinical situation commonly encountered in dentistry. This situation arises because molars have been reported to be frequently lost due to caries [1–3] in both children and adults and, moreover, because premolar aplasia is common: The prevalence of congenitally missing lower premolars has been reported to range from 2.5 to 4.0 % and is only exceeded by the absence of third molars [4–6]. Common therapeutic approaches include substitution of missing teeth using prosthodontics and/or implantology, transplantation of teeth, and orthodontic space closure [7–10]. The advantage of choosing the latter as a treatment option is that it may be applied to both (pre-)adolescent and adult patients, whereas auto-transplantation of tooth germs is considered to be sufficiently promising only in the earlier stages of development [9]. Moreover, in difficult clinical situations, such as space closure without maxillary counterbalance extraction [7], orthodontic mandibular molar protraction has been shown to be both achievable and practical, provided adequate anchorage is available [11]. While space management solutions including the use of auto-transplants or implants are basically viable methods [9, 12, 13], one shortcoming is that they require oral surgery; even more, implants require prior completion of facial growth. Therefore, despite 5 year survival rates of both auto-transplanted teeth and implants ranging from a promising 85–95 % for the former and almost 97 % for the latter [14–16], orthodontic space closure is widely accepted as being an approach that may be applied universally, independent of the subject’s age and, in addition, it may offer benefits in terms of long-term functional and periodontal conditions, without the need for surgical intervention and without artificial replacement of teeth, and also often allows for a simultaneous correction of malocclusion along with gap management.

In the context of orthodontic space closure other than uncontrolled tipping of teeth, a common problem which needs to be overcome is that of anchorage loss, as would be typical as a result of using power chains or pull-strings without adequate anchorage reinforcement [7, 10, 14, 15, 17].

Contemporary strategies for orthodontic anchorage reinforcement include the use of temporary anchorage devices (TADs) [15]. These have been shown to have an incidence of loss or loosening of mini-screws of about 19.3 % [15]. Another strategy for increasing anchorage in cases requiring lower molar protraction is the use of fixed functional appliances, such as the Herbst appliance, especially when a sagittal mandibular deficiency is apparent [18, 19].

### Study objective

**Fig. 1:**
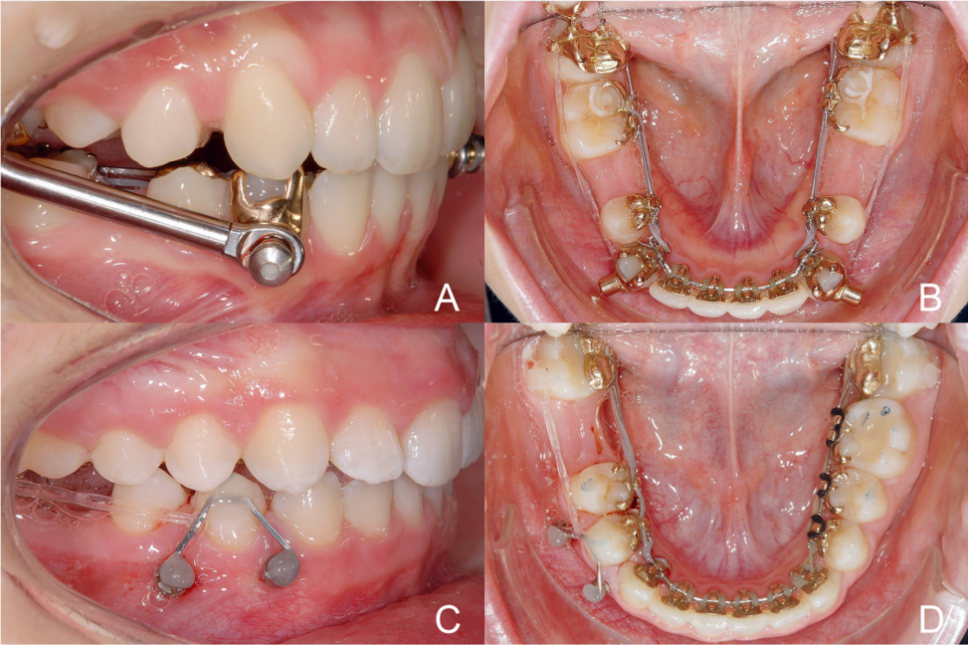
Clinical examples of the Herbst (upper row) and TAD (lower row) anchorage re-inforcements and double-cable protraction mechanics used in this study

The aim of the present study was to compare the clinical effectiveness of anchorage reinforcement of the two methods, TAD or Herbst (Fig. 1), in combination with a completely customized lingual appliance (Incognito, 3 M Top-Service für Lingualtechnik, Bad Essen, Germany) by testing the null-hypothesis of no significant difference in terms of speed of space closure (measured in mm/month) between them. Our secondary aims included descriptions and comparisons of the quality of posterior space management and treatment-related factors, such as loss of anchorage (in terms of proportions of space closure resulting from posterior protraction or anterior retraction), potential improvements in canine occlusion, and incidence of incomplete space closure.

## Subjects

Our study was a retrospective analysis of 27 subjects (males/females 12/15, 44.4:55.6 %; mean age at start of space closure 16.54 ± 1.82 years) who were treated in one orthodontic center (Prof. Dr. D. Wiechmann and colleagues, Bad Essen, Germany) between January 2003 and December 2013, with the subject inclusion criteria of

- congenitally missing or extracted lower second premolars or lower first molars,
- therapy by orthodontic molar protraction without maxillary counterbalance extraction, and
- completed treatment with a lingual appliance (Incognito, 3 M Top Service für Lingualtechnik, Bad Essen, Germany).

Patients were recruited consecutively; they were treated primarily by mini-screws from January 2003 to June 2009, and by Herbst from July 2009 to December 2013. Treatment plans were approved by one clinician (DW) prior to starting orthodontic treatment.

There was no exclusion of any subject who met the inclusion criteria regardless of later delays in treatment course due to lack of compliance, absence of tissue response, or other cause.

**Fig. 2:**
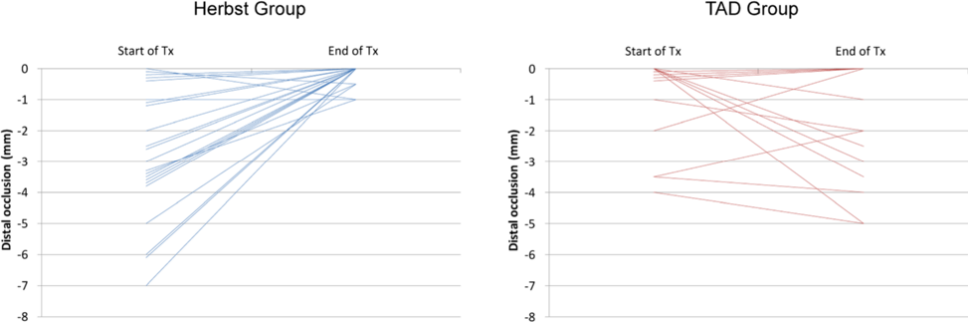
Graphic representation of improvement in distal occlusion following treatment in either of the distinctive anchorage groups

Table 1 provides details of the characteristics of the study cohort. The distribution of the initial sagittal malocclusion by Angle-Classes is documented in Fig. 2.

**Table 1:** Descriptive analysis of subjects’ ages at the time of initiating protraction (T1, years), and sex

	Valid N	Mean age (Y)	SD	Minimum	Maximum	Median
Females	15	16.74	2.19	13.417	20.99	16.34
Males	12	16.29	1.28	14.845	19.73	16.14
All Groups	27	16.54	1.82	13.417	20.99	16.14
Herbst	15	16.43	1.63	14.798	20.57	16.14
TAD	12	16.68	2.11	13.417	20.99	16.39
All Groups	27	16.54	1.82	13.417	20.99	16.14

No significant difference in subjects’ age distribution was found between male and female subjects (unpaired *t*-test, *p* = 0.51) or between the Herbst or TAD group (unpaired *t*-test, *p* = 0.74). Also, no significant difference in subjects’ sex distribution was found between the Herbst and TAD groups (Fisher’s exact test, *p* = 0.7)

## Methods

### Protraction mechanics

Based on the orthodontic anchorage strategy used, subjects were allocated to one of two groups: Group 1 (15 subjects) used a Herbst fixed functional appliance, whereas anchorage in subjects of Group 2 (12 subjects) was implemented by mini-screws (temporary anchorage devices, TAD, Fig. 1). Mini-screws were placed by Dr. Dr. A. Berens, Hannover, and/or DW. All TAD placements were approved prior to implementing protraction mechanics, by DW. Space closing by means of protraction in the Herbst group was bilateral in 7 subjects and unilateral in 8 subjects (total: 22 single situations), bilateral in 2 subjects, and unilateral in 10 subjects belonging to the TAD group (total: 14 separate situations). The mean ± SD spaces to be closed by protraction of molars was 8.0 ± 2.6 mm in the Herbst group, or 7.2 ± 2.5 mm in the TAD group. Initial mean ± SD canine distal occlusion was 2.6 ± 2.1 mm in Herbst and 1.0 ± 1.6 mm in TAD subjects.

Space closure was achieved by double-cable mechanics, in order to reduce friction resulting from arch-wire binding, as well as rotations. In addition, occlusal pads on the second molars of the lingual multi-bracket appliance helped to avoid occlusal interference by antagonistic teeth. Herbst telecopes were activated in individual step-wise increments, with a final over-correction of a sagittal discrepancy. The forces applied to the protraction of teeth by the two power chains were at the time point of implementation up to 150 cN (1.5 N) per power chain or side, *i.e.* up to a maximum of 300 cN per protraction mechanic.

### Documentation

Progress of molar protraction was documented by taking intra-oral photographs that were taken both from the top-view perspective (strictly perpendicular to the occlusal plane) and also strictly laterally, using an appropriate intra-oral mirror technique and a digital camera (D200, with Nikkor 105 mm; Nikon, Tokyo, Japan). Protraction distances were measured on these photographs at the time of insertion of the respective mechanics used for anchorage (T1), following removal of the anchorage mechanics (T2), and also at the end of lingual MB treatment (T3). Each individual photograph was separately calibrated or scaled by measuring the width of one premolar on the corresponding plaster cast and transferring the scale to the photographs (premolar width [plaster cast]/[photography]). Protraction distances measured on the photographs were multiplied by individually calculated scales. Velocity of protraction was determined by dividing the total protraction distance by the duration of protraction and expressed in mm/month.

The extent of a potential change in the canine relationship was determined by T0 and T3 photographs. A baseline value of 0 mm was assigned in cases of an Angle-Class I canine relationship (summit of upper canine’s crown corresponding with approximal contact of lower canine/first premolar). Deviations towards an Angle-Class II or III relationship were, by definition, assigned negative or positive values.

A potential loss of anchorage (in terms of proportions of space closure caused by retraction of the anterior segment) was determined by assessing the position of the lower canines relative to the summit of the upper canine’s crown as a reference, at time points T1 and T2. Similarly to the definition of distal occlusion, a deviation from baseline canine occlusion in distal direction was defined as a loss of anchorage and measured in millimeters.

### Statistical and methodological error analysis

Apart from descriptive data analysis using mean values and standard deviations, comparisons between the study groups were carried out by using repeated measures ANOVA and unpaired *t*-test. Age and sex distribution in both groups were compared using unpaired *t*-test (age) and Fisher’s exact test (sex). All tests were performed at a significance level of α = 5 % (*p* < 0.05 considered as statistical significant). All analysis were derived using SAS 9.3 (SAS Institute, Cary, NC, USA).

Intra-examiner reproduceability of measurements was determined by re-assessing photographs of 20 individual sites following an interval of 6 weeks after trial assessments using Dahlberg’s formula: [20].

$$ \mathrm{ME}=\sqrt{\frac{d^2}{2n}} $$

where *d* is the difference between single assessments and *n* the number of assessments.

The method error was 0.33 mm for measurements of the protraction distance, 0.19 mm for canine occlusion assessments, and 0.36 mm for assessing potential losses in anchorage.

### Ethical approval

This study received prior ethical approval from the University of Hannover Medical School (#1220-2011; MHH, Hannover, Germany).

## Results

### Composition of groups

There was an equal distribution in terms of subjects’ age and sex between the study groups: No significant differences were found between subjects in the Herbst or TAD groups (age, *p* = 0.7, and sex, *p* = 0.7). See Table 1 for further descriptive characteristics of the study subjects.

### Treatment-related features

**Fig. 3:**
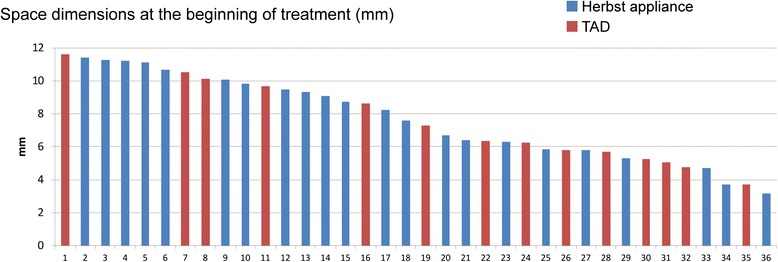
Graphic presentation of protraction distances (mm) at initiation of protraction (T1)

**Fig. 4:**
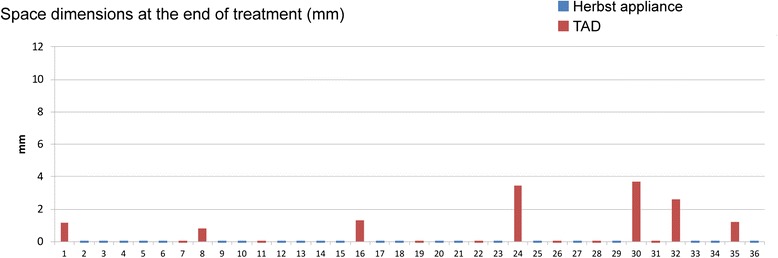
Graphic presentation of gap residues (mm) at the end of protraction and MB treatment (T3)

Mean protraction durations were 17.14 months (min/max/SD: 6.7/24.4/4.95 months) in the Herbst group and 21 months (min/max/SD: 5.44/54.8/14.01 months) in the TAD group. Initial (T1) protraction distance had a mean value of 8 mm in the Herbst (min/max/SD: 3.1/11.4/2.6 mm), and 7.2 mm (min/max/SD: 3.7/11.6/2.5 mm) in the TAD group (Fig. 3). Complete space closure following anchorage mechanical removal (T2) was achieved in 68.2 % [28.57 %] of the Herbst [TAD]-treated subjects (average remaining gap dimensions: 0.5 mm (min/max/SD: 0/3.2/1 mm) [TAD: 2 mm (min/max/SD: 0/7.3/2.3 mm)]. At the time of removal of the lingual multi-bracket appliance (T3), complete space closure was accomplished in all of the Herbst-treated subjects, but only in 50 % of the TAD group (Fig. 4). Remaining gaps at T3 in the TAD group had a mean dimension of 1 mm (min/max/SD: 0/3.5/1.3 mm).

**Fig. 5:**
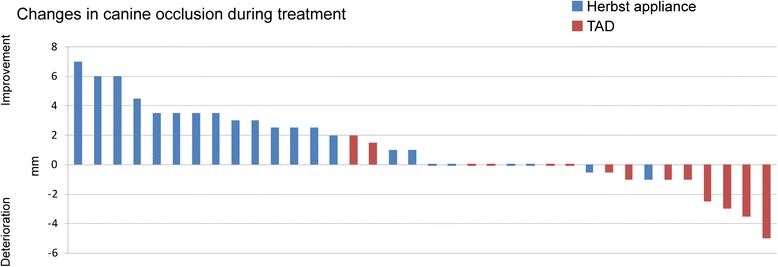
Graphic representation of changes in canine relations in the two groups of Herbst or TAD reinforced subjects. There was an improvement in canine occlusion in 90.9 % of Herbst-treated and 14.29 % of TAD-treated subjects

On average, initial (T0) distal occlusion was −2.7 [−1] mm (min/max/ SD: 0/-7/2.1 mm [0/-4/1.56 mm]) in the Herbst [TAD] group. The canine relationship was improved towards class I occlusion in 90.9 % [14.29 %] of Herbst [TAD] patients, by a mean 2.4 mm (min/max/SD: 0/-2/0.5 mm) in Herbst-treated cases until T3 (Fig. 5); the canine relationship deteriorated as a result of loss of anchorage in two of the Herbst-treated subjects, to an extent of 2 mm each. In contrast, deterioration in the canine relationship was seen in 57.1 % or *n* = 8 of TAD-treated subjects, to an extent of a mean 1 mm (min/max/SD 0/-5/1.89 mm) at T2 (Fig. 5). There was a loss of five mini-screws in four subjects (36.7 % of sites, or 33.3 % of subjects).

### Velocity of molar protraction (in mm/month)

**Fig. 6:**
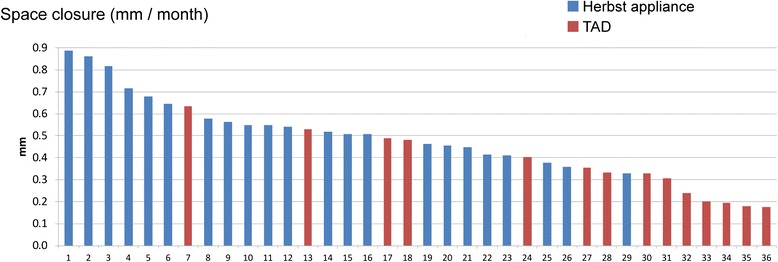
Graphic representation of mean velocity of protraction (mm/month) at initiation of protraction (T1). See also Table 2 for details

Table 2 and Fig. 6 provide a descriptive breakdown of velocity of protraction for both groups, as well as each single (left or right) protraction site.

**Table 2:** Descriptive breakdown of velocity of protraction for both groups and for each individual protraction site

	Valid protraction sites (N)	Mean protraction distance (mm/month)	SD	Minimum	Maximum	Median
Herbst	22	0.51	0.19	0.17	0.89	0.51
TAD	14	0.35	0.15	0.17	0.63	0.33
All Groups	36	0.45	0.19	0.17	0.89	0.45

Repeated measures ANOVA with the subject as the repeated factor and the protraction method [Herbst; TAD] and location [left;right] as fixed factors revealed a significant difference (*p* = 0.008) between both methods, in terms of duration of protraction, with an increased molar protraction velocity in Herbst-treated subjects, but no significant differences in terms of duration of left- or right-sided protraction (Table 3).

**Table 3:** Repeated measures ANOVA with the subject as repeated factor revealed a significant difference between both methods in terms of velocity of protraction

Effect	*p*-value
Method (Herbst; TAD)	0.008
Site location (right/left)	0.8
Interaction Method * Site location	0.2

There was no significant effect of left- versus right-sided site location

In addition, in those subjects who received a bilateral molar protraction, values of left and right-sided protractions were averaged to one value per subject and used as a basis for a comparison of protraction methods (Table 4). Accordingly, there was also a significant difference between both methods in terms of duration of protraction (unpaired *t*-test, *p* = 0.013).

**Table 4:** Comparison of protraction effectiveness following averaging of values for left- and right-sided protractions in those subjects who received a bilateral molar protraction

	Valid subjects (N)	Mean mm/month	SD	Minimum	Maximum	Median
Herbst	15	0.53	0.19	0.19	0.89	0.46
TAD	12	0.36	0.15	0.18	0.63	0.34
All Groups	27	0.45	0.19	0.18	0.89	0.34

There was a significant difference between both methods in terms of duration of protraction (unpaired *t*-test, *p* = 0.013)

## Discussion

While the implementation of mini-screw or TAD-supported reinforcement during orthodontic protraction of teeth in edentulous sites has been widely accepted as a concept for creating maximum anchorage [21–23], the common downsides of this technique are a potential loosening or tilting of TADs following loading [16, 24], potential collateral damage to roots during inter-radicular TAD placement [16, 25, 26], and, in clinical situations in which either contra-indications apply or patients or their guardians object to TAD placement for personal reasons. Moreover, proportions of (mid-palatal) TAD failure have been reported to be much more pronounced in patients who are aged 15 or younger, with success rates of about 71 %, indicating a need for offering alternative treatment approaches [27]. Therefore, the use of fixed, functional appliances such as the Herbst appliance, which are readily available in orthodontic surgeries, suggests itself to be a viable treatment option for gaining anchorage during mandibular molar protraction. Our study objective of comparing the clinical effectiveness of the two treatment approaches in terms of providing maximum anchorage during lingual orthodontic molar protraction therefore seems justified.

### Assessment method

Great care was taken to achieve a high degree of standardization during photographic documentation and study measurements: Lateral intra-oral photographs were made strictly perpendicular to the posterior teeth using intra-oral mirrors and cheek-holders. Based on the ‘true’ dimensions assessed by one premolar of the corresponding plaster cast, individual scaling of each of the photographs facilitated assessment of protraction distances based on those calibrated digital pictures. An assessment of method error produced results with an acceptable level of reproducibility.

### Null-hypothesis

The null-hypothesis of no significant difference in terms of speed of space closure (measured in mm/month) between molar protraction mechanics using either TAD support or Herbst appliances as anchorage was rejected: molar protraction was accomplished significantly earlier in Herbst-treated cases (*p* = 0.008).

### Clinical effectiveness and occlusal side-effects

In view of the significantly increased molar protraction performance when using Herbst-supported anchorage, one has to take into account the fact that early losses of single mini-screws occurred on five occasions of the 14 sites, making it necessary to continue space management without TAD anchorage. The proportion of TAD failures corresponds to the value of 19.3 % reported in the literature for mandibular mini-screw loosening [16], which seems to be influenced by a variety of biological co-factors [21, 22, 24]. Midterm changes in treatment plans are not considered to be feasible, for obvious reasons, and as orthodontic mini-screws show an increased tendency to fail after 4–5 months following load application [28], it is common to continue treatment in such cases without renewed TAD anchorage support. This seems to be a clear drawback of the TAD technique, especially in situations requiring time-consuming and laborious orthodontic space closure at sites which may have been edentulous for long periods. This has to be considered as a factor that decreases overall molar protraction velocity, and the indication for TADs may be constrained in situations of mandibular molar protraction.

Improvement in canine occlusion was found to be significantly increased in subjects belonging to the Herbst group, which fulfils expectations, as the Herbst appliance was basically designed as a concept for sagittal mandibular advancement [18, 29]. Previous research has indicated that mandibular incisor proclination has to be considered to be a typical side-effect of the use of Herbst appliances [18]. Although this side-effect seems to be reduced in cases treated by a Herbst appliance in combination with a completely customized lingual MB appliance, this side-effect is clearly to be seen as a factor that provides some additional anchorage or counter-force for cancelling side-effects of forces used for protraction of molars [30, 31]. It also explains the pronounced increase (90.9 % of situations, mean improvement 2.61 mm) in improvement in the canine relation.

**Fig. 7:**
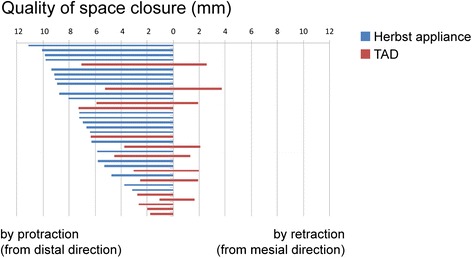
Graphic representation of quality of space closure (by protraction, or by protraction and distalization of anchorage teeth) or mean anchorage loss. In the Herbst group, space closure was achieved solely by protraction of posterior teeth and with a success rate of complete gap closure in 100 % in all of the 22 space closure sites, with a mean protraction distance of 7.4 ± 2.2 mm. In the TAD group, space closure was achieved in 76.9 % of sites (mean distance 4 ± 2.1 mm) from distal direction (protraction), and in 23.1 % (mean distance 1.2 ± 1.2 mm) from mesial direction, indicating a loss of anchorage. Space closure in the TAD group was incomplete in 50 % of cases, with gap residues of a mean 1 mm

Other than early failure of mini-screws, deterioration of the canine relationship towards distal occlusion or losses of anchorage of a mean 1 mm (SD: 1.2 mm) to a maximum of 5 mm (Figs. 2 and 7) may -to some extent- reflect a tilting of mini-screws by about 1–1.5 mm, as has been reported in the literature [32].

Therefore, the use of a Herbst appliance as an anchorage device in subjects requiring molar protraction along with maintenance of an Angle Class I occlusion or even an improvement in distal occlusion is indicated rather than the use of TAD reinforcement.

### Velocity of protraction

The primary aim of the study was a comparison of the clinical effectiveness of either TAD supported - or Herbst-reinforced anchorage during molar protraction in subjects treated with lingual multi-bracket appliances. Overall, velocity of protraction (mm/month) was found to be 0.51 in Herbst treated subjects, and 0.35 in TAD-anchored situations (Table 2), which is in agreement with a majority of published studies [23, 33], while other authors reported up to 0.76 mm/month on average, using open coils springs and a balanced anchorage between the six anterior teeth and the second premolar and first molar posteriorly [34]. Generally, space closure following extraction does not take place at a linear rate, but may be up to 0.86 mm during the first months, and is known to subsequently continue at a slower rate of about 0.3 mm/month [35].

Complete space closure following completion of MB treatment was accomplished in only 50 % of subjects with TAD anchorage, with remaining gaps of 1 mm on average (Fig. 4), while complete space closure was achieved in all of the Herbst-reinforced protraction sites (Fig. 4). This result is in agreement with other reports, which also found gap residues following orthodontic space closure with a mean of 1.5 mm in 46 % of subjects with bilateral premolar aplasia treated by push-and-pull mechanics [36].

This study compared two competing treatment alternatives for gaining anchorage during space closure (lingual appliance plus Herbst, or lingual appliance plus TAD). The findings have a limitation concerning generalisability in that they were achieved with a lingual Herbst appliance that is separated from the lingual multi-bracket appliance, in contrast to Herbst derivates that are attached to the archwire, or to those using casted splints. Tooth movement along archwires is unimpeded here, while it may not be so with conventional Herbst appliances using casted splints and labial fixed orthodontic appliances.

## Conclusion

The following conclusions can be drawn regarding the quality of molar protraction in subjects treated with lingual MB appliances:

- The use of a Herbst appliance as an anchorage reinforcement provides increased anchorage control, as protrusive forces of the appliance are effective in cancelling the distalizing side-effects of protraction forces.
- Therefore, Herbst-reinforced space closure was found to be faster and judged to be more reliable compared to TAD anchorage.
- Patients requiring simultaneous space closure by molar protraction and correction of distal occlusion may benefit from using Herbst appliances for anterior segment anchorage reinforcement rather than TAD anchorage.
